# DPCR-SLAM: A Dual-Point-Cloud-Registration SLAM Based on Line Features for Mapping an Indoor Mobile Robot

**DOI:** 10.3390/s25175561

**Published:** 2025-09-05

**Authors:** Yibo Cao, Junheng Ni, Yonghao Huang

**Affiliations:** School of Artificial intelligence, South China Normal University, Foshan 528225, China; vacuame@163.com (J.N.); reancolstark@163.com (Y.H.)

**Keywords:** Simultaneous Localization and Mapping, laser radar, optimization, Iterative Closest Point, real-time systems

## Abstract

Simultaneous Localization and Mapping (SLAM) systems require accurate and globally consistent mapping to ensure the long-term stable operation of robots or vehicles. However, for the commercial applications of indoor sweeping robots, the system needs to maintain accuracy while keeping computational and storage requirements low to ensure cost controllability. This paper proposes a dual-point-cloud-registration SLAM based on line features for the mapping of a mobile robot, named DPCR-SLAM. The front-end employs an improved Point-to-Line Iterative Closest Point (PLICP) algorithm for point cloud registration. It first aligns the point cloud and updates the submap. Subsequently, the submap is aligned with the regional map, which is then updated accordingly. The back-end uses the association between regional maps to perform graph optimization and update the global map. The experimental results show that, in the application scenario of indoor sweeping robots, the proposed method reduces the map storage space by 76.3%, the point cloud processing time by 55.8%, the graph optimization time by 77.7%, and the average localization error by 10.9% compared to the Cartographer, which is commonly used in the industry.

## 1. Introduction

Simultaneous Localization and Mapping (SLAM) serves as the cornerstone for autonomous navigation in indoor cleaning robots. To ensure long-term stable operation, SLAM systems must have high-precision mapping capabilities and global consistency. Recent research has increasingly focused on precision enhancement, with significant advances in geometric-aware 3D point cloud processing for high-precision applications [1]. High-precision mapping and global consistency are essential for long-term operation. However, commercial viability also requires balancing algorithmic performance and hardware cost efficiency. This trade-off is often overlooked in academic research but remains critical for mass production [2]. For indoor robots, it is essential to develop solutions that precisely meet their localization accuracy requirements while maintaining cost-effectiveness.

Existing SLAM solutions face a trilemma between computational complexity, storage efficiency, and mapping accuracy. Filter-based approaches like Gmapping [3] suffer from particle degeneracy in large environments, while graph optimization methods [4] require substantial memory for submap storage. Although ICP variants such as PLICP [5] improve registration accuracy, their computational load remains prohibitive for the low-cost embedded processors typically used in commercial cleaning robots. This mismatch between algorithmic demands and hardware constraints leads to either compromised mapping quality or unsustainable production costs—a fundamental barrier to market adoption.

This paper proposes DPCR-SLAM, a dual-point-cloud-registration framework specifically designed for indoor mobile robots.Typical indoor environments contain abundant linear structural features, so we leverage structural line features to enhance environment mapping. To be more specific, our method uses an improved Point-to-Line Iterative Closest Point (PLICP) algorithm to register point clouds from the most recent period to obtain a submap, divides the map space into relatively independent regional maps at certain distance intervals, and then obtains regional maps through the registration of submaps. The back-end performs graph optimization based on the relationships between regional maps. To this end, the main contributions of this paper can be summarized as follows:

1. Designs a system framework based on double registration, strengthening the data association between reference point clouds and target point clouds, improving the positioning accuracy and mapping effect of indoor cleaning robots.
2. Adopts an improved Point-to-Line ICP algorithm leveraging structural regularities in indoor environments, achieving faster convergence than conventional PLICP through directional error constraints.
3. Performs graph optimization based on data between regional maps, with lower dimensionality of error equations, thus reducing graph optimization processing time.
4. Only regional maps are needed to store as nodes of the graph structure, reducing the storage space required for maps.

The rest of this paper is structured as follows: Section 2 introduces related work. Section 3 provides the entire process and details of the method. Section 4 analyzes the effect of regional width. Section 5 shows the experimental results. Section 6 summarizes the work and plans for future work.

## 2. Related Work

### 2.1. Iterative Closest Point

In the LiDAR sensing domain, current odometry pipelines typically apply some form of ICP to estimate poses incrementally [6]. The Iterative Closest Point (ICP) algorithm approximates the true value through iterative rigid body transformations. This algorithm does not require feature extraction from the point cloud, but it does require a good initial position and has high requirements for point cloud overlap. Censi [5] proposed the Point-to-Line ICP (PLICP) algorithm, which uses the lines formed by adjacent points as matching objects instead of individual points, improving the matching accuracy. However, this algorithm has poor robustness when the initial position is not ideal. Hong et al. [7] introduced velocity changes into the ICP process to address motion distortion, calling it the Velocity Update ICP (VICP) algorithm. Yang et al. [8] proposed the Global Optimal ICP (Go-ICP) algorithm, which integrates local matching into a branch-and-bound scheme, avoiding dependence on the initial position. Alismail et al. [9] designed the CICP algorithm to correct the distortion caused by sensor motion during the point cloud registration process, improving the algorithm’s accuracy and robustness. The above methods are based on point scanning, which can easily lead to local convergence and do not consider sensor noise [10].

### 2.2. Filter-Based SLAM

Before the popularity of graph optimization, Bayesian filtering ideas were the preferred choice for mapping in the SLAM field. Researchers initially adopted Kalman Filter (KF)-based methods for mapping. KF methods estimate the system states within linear systems, resulting in large deviations over time [11]. To address this, the Extended Kalman Filter (EKF) method uses Taylor expansion to linearize non-linear relationships, selecting low-order terms for calculation to approximate linearized results [12]. However, the EKF method is computationally expensive and difficult to apply in large-scale environments [13]. The Unscented Kalman Filter (UKF) treats system states and measurement data as samples, using weighted coefficients to sum them up and approximate true values, avoiding the accumulation of non-linear errors [14]. The Extended Information Filter (EIF) method describes system states using information matrices and generates sparse matrices through weak associations between these matrices, reducing computational consumption [15]. Thrun et al. proposed the Particle Filter (PF) method, which assigns different weights to particles and adjusts system states through particle summation to approximate true probability distributions. However, as the working time increases, the number of particles also increases, leading to large computational and storage consumption [16].

### 2.3. Graph Optimization SLAM

Currently, the most commonly used robot mapping method is Graph Optimization (GO), which solves SLAM problems by establishing graph structures [17]. The algorithm’s front end uses the EKF method, PF method, or other methods to construct maps, update robot poses, and build graph structures. The back end then solves and optimizes the graph structure.

Lu et al. applied a graph optimization framework to achieve mapping and localization, using non-linear least squares to optimize cumulative errors in the mapping process. This method ignores the sparsity of the system, and the solving method has high computational costs, thus greatly limiting real-time performance [18]. Gutmann et al. proposed Local Registration and Global Correlation (LRGC), an algorithm that successfully achieves mapping and localization in environments with large periodic structures; however, it relies on pose consistency estimation and map correlation, performing poorly in non-periodic structured environments [19]. Bosse et al. constructed the ATLAS framework, using Kalman filtering at the bottom layer and global optimization methods at the top layer to align local maps. This method provided a new approach for graph structure construction, but due to its complex structure, it has not been widely applied [20]. Kaess et al. proposed incremental smoothing and mapping (iSAM). This method incrementally optimizes nodes using least squares during robot operation, offering good real-time performance; however, the algorithm has high computational requirements and poor global consistency [21]. K. Konolige et al. proposed Karto SLAM, using sparse Cholesky decomposition to solve linearized equations, called Sparse Pose Adjustment (SPA). This method greatly improved the algorithm’s real-time performance, making real-time mapping possible in large-scale environments [22]. However, the algorithm heavily relies on odometry and lacks loop closure detection [23]. Kohlbrecher et al. proposed Hector-SLAM, using the Gauss–Newton method for front-end data association and frame-to-map scan matching. However, this method lacks back-end optimization, is sensitive to initial values, and has no loop closure detection [24]. Hess et al. proposed Cartographer [25], which constructs local maps by fusing multi-sensor information and uses frame-to-submap scan matching for loop closure detection. Cartographer can achieve mapping in large indoor environments but has high requirements for computational speed and storage space, limiting its real-time performance [26]. Newcombe et al. [27] proposed Kinect Fusion for RGB-D data. This method fuses all depth data from sensors into a depth map represented by a globally truncated signed distance function (TSDF), using a coarse-to-fine ICP method to track real-time depth frames while obtaining the current sensor pose. KinectFusion achieved good mapping results in indoor environments, but it consumes extensive computational and storage resources and is easily affected by environmental light [28]. Recent advances include ORB-SLAM3 [29], which generalized visual-inertial SLAM with multi-map management, and LeGO-LOAM [30], which introduced lightweight ground-optimized LiDAR odometry. The above methods have achieved some success in practice, but in the context of indoor cleaning robots, they still face several challenges, including high computational load, significant storage requirements, and relatively large errors.

## 3. System Description

### 3.1. Workflow

**Figure 1:**
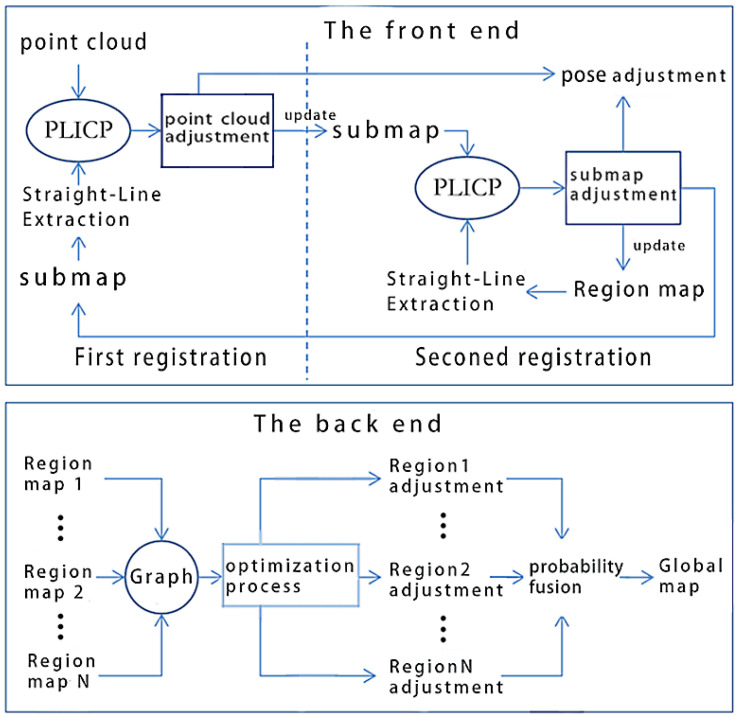
System framework.

Figure 1 shows the framework of the proposed SLAM system.

As shown in Figure 1, the proposed method applies a graph optimization model, divided into front-end and back-end parts.

The front end includes two registration processes.

- First registration: Using the improved PLICP, the point cloud and submap are registered. Then, the point cloud and robot pose are adjusted based on the registration results. The adjusted point cloud will be used to update the submap. We establish the submap from the most recent N frames of point clouds. At the start of the operation, when no submap exists, the point cloud is directly used to initialize the submap.
- Second registration: Align the updated submap and regional map by using the improved PLICP. Based on the registration results, the submap and robot pose are adjusted. The regional map is a grid map formed by fusing submaps within a region. These regions are obtained by dividing the workspace into relatively independent sections at a set distances. In the initial stage of the robot entering a region, the regional map has not yet been established, so the submap is directly used to update the regional map.

The back end primarily focuses on managing the graph optimization process. In the proposed method, the robot poses corresponding to regional maps serve as vertices in the graph structure, while data associations between regional maps constitute the edges. The graph structure is solved using the Gauss–Newton algorithm, and the results are used to rectify both the regional maps and robot poses. Subsequently, the adjusted regional maps are integrated through probabilistic fusion to generate the global map.

### 3.2. Improved PLICP

#### 3.2.1. Steps

Traditional point-line matching algorithms form a local straight line with two proximity points, then construct the error function based on the distance from the target point to the local line. Our work proposes an improved PLICP algorithm that constructs the target error function based on the distance from the target point to the line feature. The algorithm steps are as follows:

(1) Use the RM-Line [31] algorithm to extract line features from the grid map. Mainly because, compared to the state-of-the-art algorithms, this algorithm has better performance on 2D indoor grid maps.
(2) Set the iteration count k = 0, the initial rotation $\theta_{k}=0$, and the initial translation $t_{k}=\left\{0,0\right\}$.
(3) Search for matching point pairs. Let the reference point cloud be $Q=\{q_{j},j=1,2,..,M\}$ and the target point cloud be $P^{k}=\left\{p_{i},i=1,2,..,N\right\}$. For each target point $p_{i}$, search for the nearest free reference point $q_{j}$. Calculate the foot of the perpendicular from $p_{i}$ to the line feature as the matching point $q_{j}^{i}$. Record the matching point pair as $C_{i}^{k}=\left\{\left(p_{i},q_{j}^{i}\right)\right\}$.
(4) Repeat step 3 to traverse all target points.
(5) Solve the rigid body transformation for the matching point pairs $C^{k}$, where the rotation angle is $\theta_{k}$ and the translation is $t_{k}$.
(6) Rotate and translate the target point cloud, as shown in Equation (1):
  (1) $$P^{k+1}=R\left(\theta_{k}\right)P^{k}+t_{k}$$
  where $P^{k}$ is the target point cloud before the kth iteration, $R(\theta_{k})$ is the rotation transformation, and $t_{k}$ is the translation transformation.
(7) Update the error function, as shown in Equation (2):
  (2) $$J\left(Q,C_{k}\right)=\;\sum_{i=1}^{N}{\left(p_{i}^{k+1}-q_{j}^{i}\right)}^{2}$$
  where $p_{i}^{k+1}$ is the ith data point in the target point cloud after the kth iteration and transformation, N is the number of points, and $q_{j}^{i}$ is the matching point of $p_{i}^{k}$.
(8) Repeat steps 3–7 until the error falls below the threshold or the maximum number of iterations is reached.

#### 3.2.2. Comparison with Conventional PLICP Algorithms

**Figure 2:**
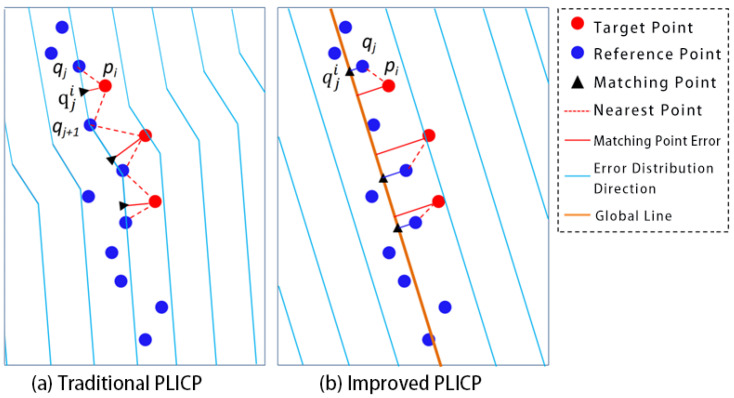
Point cloud matching and error distribution of two algorithms.

Figure 2 shows the difference between the proposed improved PLICP algorithm and the traditional PLICP algorithm. In Figure 2, the black triangle represents the matching point corresponding to the target point during the rigid body transformation. $q_{j}$ and $q_{j+1}$ are the nearest and second-nearest reference points to the target point $p_{i}$, respectively.

In the traditional PLICP algorithm (Figure 2a), the target point $p_{i}$ uses its perpendicular foot $q_{j}^{i}$ on the line $\;{<q}_{j},q_{j+1}>$ as the matching point. The distance from pi to the line ${<q}_{j},q_{j+1}>$ is treated as the error term. As can be seen from the blue lines in the figure, the error distribution directions corresponding to each data point in the reference point cloud are inconsistent, depending on the relative positions of adjacent points.

As shown in Figure 2b, in the proposed improved PLICP algorithm, the target point $p_{i}$ employs the orthogonal projection $q_{j}^{i}$ of the nearest point $q_{j}$ on the line feature as the matching point, and the distance from it to the line is the error term. Within the local range shown in the figure, the error distribution directions corresponding to the data points in the reference point cloud are all consistent with the line feature, which is closer to the true contour position. This feature-level representation reduces the influence of outliers and measurement errors common in indoor environments, inherently smooths local random noise effects, and enhances the robustness of the registration process. In addiction, since the method depends primarily on geometric consistency rather than point density, it is expected to remain applicable under different LiDAR resolutions

### 3.3. The First Registration Process

**Figure 3:**
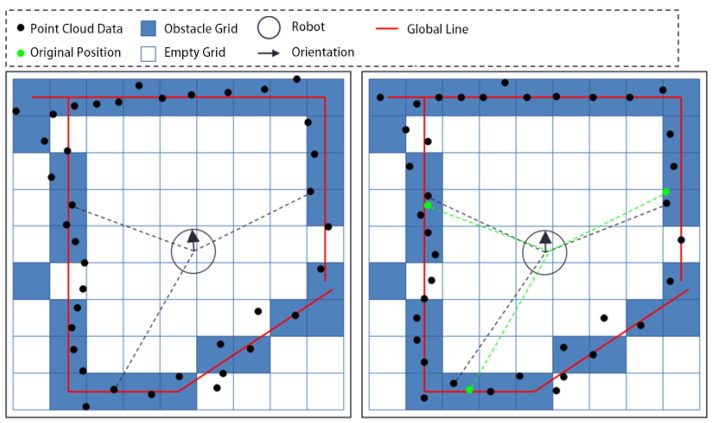
The first registration.

The proposed method fuses a segment of time’s range point clouds to obtain a submap. Due to factors such as grid precision, motion errors, control errors, and measurement errors, there is inevitably some deviation between the point cloud and the submap. To address this, a point cloud matching method is used to register the point cloud and the submap, a process referred to as the first registration, as shown in Figure 3.

Figure 3 shows that the distance between the point cloud and line features is large before registration, and the distance becomes smaller after registration. As described in Section 3.2, the registration process involves rotating and translating the point cloud, and the same operations are applied to the robot’s pose.

The first registration process is described as follows.

(1) Extract lines from the submap at regular intervals: The improved PLICP algorithm requires extracting line features from the submap. However, in indoor environments most obstacles are relatively stable and the grid map has statistical properties. Therefore, the line features in the submap change relatively slowly. Based on this, the first registration process extracts line features from the submap every $K_{1}$ frames to reduce computational complexity.
(2) Extract obstacle grid cells from the submap and use their center points as data points to form a reference point cloud.
(3) Execute the improved PLICP algorithm.
(4) Adjust the point cloud and robot pose based on the matching results.
(5) Update the submap grid using the adjusted point cloud.

### 3.4. The Second Registration Process

The proposed method divides the workspace into several relatively independent regions at a set distance interval and fuses the robot’s submap within the region to obtain a regional map. At the beginning of the robot’s work, the regional map has not been established and the submap is directly used to update the regional map. After the regional map is established, the improved PLICP method is applied to align the submap and the regional map and then update the regional map with the adjusted submap.

#### 3.4.1. Regional Division

**Figure 4:**
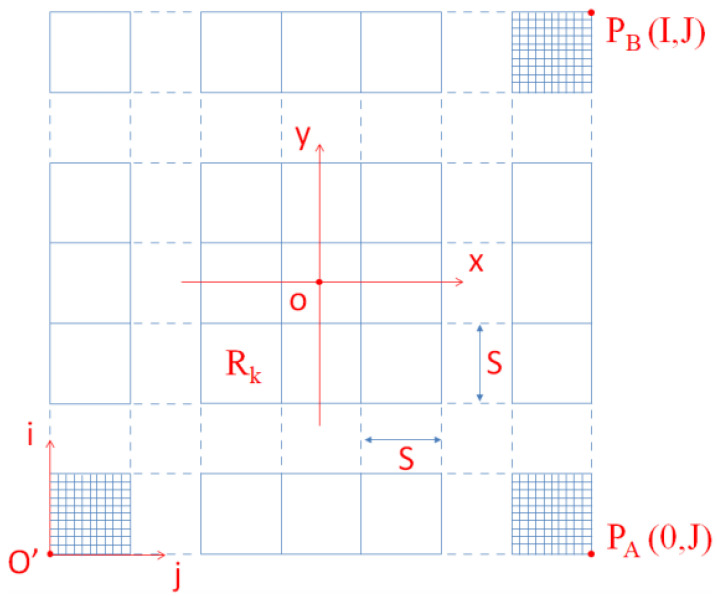
Division of regions.

The proposed method divides the workspace into several relatively independent regions at a set distance interval, as shown in Figure 4.

In Figure 4, xoy is the robot’s world coordinate system and $io^{\prime }j$ is the grid coordinate system corresponding to the global map. The conversion relationship between the robot’s grid coordinates and world coordinates is shown in Equation (3).

(3) $$\left\{\begin{array}{c} ri=round\left(\frac{y}{w_{g}}\right)+\frac{I}{2}\\ j=round\left(\frac{x}{w_{g}}\right)+\frac{J}{2} \end{array}\right.$$

In Equation (3), (x,y) are the world coordinates of the data point, (i,j) are the grid coordinates corresponding to the data point, $w_{g}$ is the set grid width, I is the number of grid cells in the i direction, and J is the number of grid cells in the j direction.

The region width S can be obtained from Equation (4).

(4) $$S=N*w_{g}\;\;,\;0<N<\min\left(I,J\right)$$

In Equation (4), $w_{g}$ is the grid width and *N* is the set multiple.

(5) $$R_{k}=fix\left(\frac{i}{N}\right)\times \frac{J}{N}+fix\left(\frac{j}{N}\right)$$

The regional number $R_{k}$ can be obtained by Equation (5), where (i,j) is the grid coordinate, *N* is the number of grids in a region, and *J* is the number of grids in the *j* direction of the grid space.

#### 3.4.2. Registration of Submap and Regional Map

**Figure 5:**
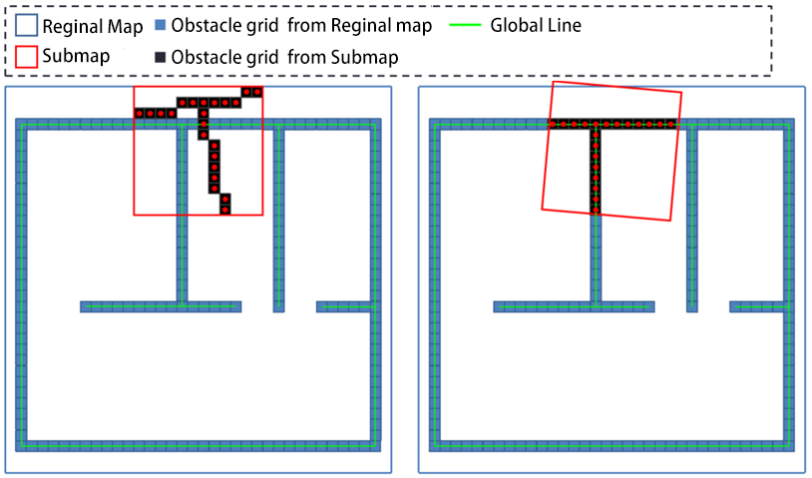
The second registration.

The registration of the submap and regional map is shown in Figure 5.

As shown in Figure 5, the submap is obtained by statistical analysis of the point cloud data from recent frames, and its coverage range is relatively small. The regional map is obtained by fusing the submaps obtained by the robot during its movement in the region, and its coverage range is relatively large.

The second registration process is described as follows:

(1) Extract lines from the regional map at regular intervals. Similar to the first registration, the line features in the regional map change more slowly than those in the submap. In the second registration process, extract lines from the regional map every $K_{2}$ frames, and $K_{2}>K_{1}$.
(2) Scan the obstacle grids from the submap and use their center points as data points to form the target point cloud; scan the obstacle grids from the regional map and form a reference point cloud with their center points.
(3) Execute the improved PLICP algorithm.
(4) Adjust the submap and robot pose based on the matching results.
(5) Update the regional map by using the adjusted submap and the target point cloud.

### 3.5. Region-Based Graph Optimization Method

At the back end of the method, graph optimization is performed based on the regional maps.

The basic idea of the region-based graph optimization method is as follows: First, establish several relatively independent regional maps during the robot’s operation. Then, build the graph optimization structure based on the data association between regional maps and construct the objective function accordingly. Next, solve the objective function with the system’s minimum error as the convergence condition. Finally, adjust the nodes in the graph structure based on the solution results.

**Figure 6:**
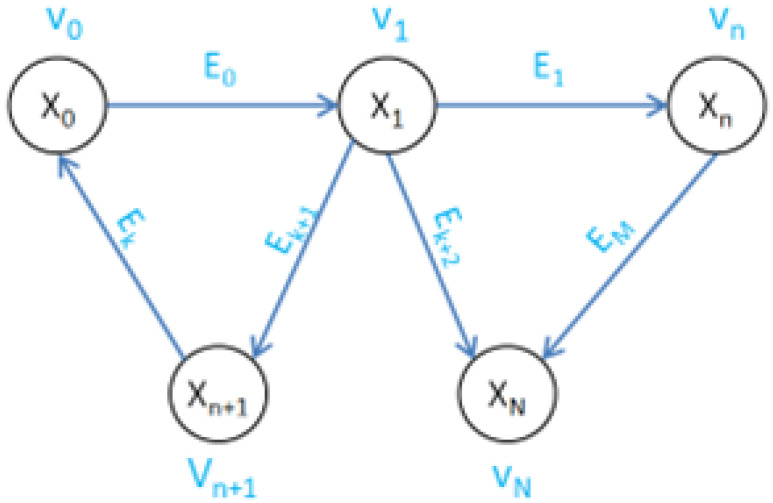
Graph structure used in proposed mapping algorithm.

The graph structure based on regional maps is shown in Figure 6.

In Figure 6, $v_{n}$ is the vertex, $X_{n}$ is the variable to be solved, $E_{k}$ is the edge, *N* is the number of vertices, and *M* is the number of edges. The vertex $v_{n}$ contains two parts of information: the point cloud $C_{n}$ and the robot’s pose ${Pos}_{n}$. Among them, $C_{n}$ is the obstacle point cloud extracted from the regional map $R_{m}$ to which the vertex belongs; ${Pos}_{n}={\left[x_{n},y_{n},\theta_{n}\right]}^{T}$ is the robot’s pose when $R_{m}$ is established. The selection method of the regional map $R_{m}$ to which the vertex $V_{n}$ belongs will be introduced in the next part. The variable to be solved $X_{n}$ is the robot’s pose contained in the vertex $v_{n}$, denoted as $X_{n}={Pos}_{n}={\left[x_{n},y_{n},\theta_{n}\right]}^{T}$.

In the graph structure, all edges are directed binary edges, meaning each edge connects two vertices, directed from the starting point $v_{i}$ to the ending point $v_{j}$. The edge $E_{k}\left(v_{i}\to v_{j}\right)$ describes the error function between $v_{i}$ and $v_{j}$ based on $v_{i}$, as shown in Equation (6).

(6) $$E_{n}\left(x_{i},x_{j}\right)=Z_{ij}^{-1}\cdot \Omega_{ij}\cdot \hat{Z_{ij}}$$

In Equation (6), $X_{i}={\left[x_{i},y_{i},\theta_{i}\right]}^{T}$ is the variable to be optimized at vertex $v_{i}$; $X_{j}={\left[x_{j},y_{j},\theta_{j}\right]}^{T}$ is the variable to be optimized at vertex $v_{j}$; $\Omega_{ij}$ is the information matrix; $\hat{Z_{ij}}$ is the estimated pose transformation matrix from $X_{j}$ to $X_{i}$; and $\hat{Z_{ij}}$ can be obtained from Equation (7).

(7) $$\left\{\begin{array}{c} \begin{array}{c} ll\hat{Z_{ij}}=\left[\begin{array}{cc} {R1}_{ij} & {T1}_{ij}\\ 0 & 1 \end{array}\;\right]\; \end{array}\\ {R1}_{ij}=\left[\begin{array}{cc} cos(\theta_{j}-\theta_{i}) & -sin(\theta_{j}-\theta_{i})\\ sin(\theta_{j}-\theta_{i}) & cos(\theta_{j}-\theta_{i}) \end{array}\;\right]\;\\ {T1}_{ij}=\left[\begin{array}{cc} cos(\theta_{i}) & sin(\theta_{i})\\ -sin(\theta_{i}) & cos(\theta_{i}) \end{array}\right]\left[\begin{array}{c} x_{j}-x_{i}\;\\ y_{j}-y_{i} \end{array}\right] \end{array}\right.$$

In Equation (7), $Z_{ij}$ is the observation equation, which is the transformation matrix from point cloud $C_{j}$ to point cloud $C_{i}$ in this paper’s graph structure, where $C_{i}$ and $C_{j}$ are the point clouds contained in vertices $v_{i}$ and $v_{j}$, respectively. $Z_{ij}$ is shown in Equation (8).

(8) $$\left\{\begin{array}{c} \begin{array}{c} llZ_{ij}=\left[\begin{array}{cc} {R2}_{ij} & {T2}_{ij}\\ 0 & 1 \end{array}\;\right] \end{array}\\ {R2}_{ij}=\left[\begin{array}{cc} cos(\Delta \theta ) & -sin(\Delta \theta )\\ sin(\Delta \theta ) & cos(\Delta \theta ) \end{array}\;\right]\;\\ {T2}_{ij}=\left[\begin{array}{c} \Delta x\\ \Delta y \end{array}\right] \end{array}\right.$$

Based on Equation (8), we can obtain the objective function for graph optimization, as shown in Equation (9), where *N* is the number of edges in the graph structure.

(9) $$F\left(X\right)=\min_{X}\sum_{n=1}^{N}{\left|\left|e_{n}\left(X_{i},X_{j}\right)\right|\right|}^{2}=\min_{X}\sum_{ij}Z_{ij}^{-1}\cdot \Omega_{ij}\cdot \hat{Z_{ij}}$$

Constructing the graph structure based on regional maps is event-driven and invoked only when the robot leaves a region and a new regional map is established. The process is described as follows:

(1) Upon the robot leaving region m, incorporate point cloud $C_{n}$ and pose ${Pos}_{n}$ corresponding to the regional map $R_{m}$ to the graph structure as the initial vertex $V_{0}$.
(2) Examine the correlation between other regional maps $R_{i}$ and $R_{m}$. If the conditions are satisfied, the point cloud and pose corresponding to $R_{i}$ will be incorporated into the graph structure as a new vertex $V_{n}$. Form an edge between $V_{0}$ and $V_{n}$, where the vertex with the earlier regional map establishment time is used as the starting point of the edge and the later one as the endpoint. The following parameters are utilized to evaluate the correlation of regional maps.
  - RegDis: The distance between the centers of the regions on the map. Due to the limitations of sensor measurement distance, the robot’s detection range within each region is limited. When the centers of two regions are far apart, the correlation between the corresponding regional maps becomes very small or non-existent. The center distance of two regional maps forming an edge should satisfy
    (10) RegDis<=S∗α
    where *S* is the region width, α is a proportional coefficient, and α≥1.
  - MatchRate: Regional map matching rate. After matching point clouds from two regional maps, we can obtain the matching degree of the two maps, as shown in Equation (11).
    (11) $$MatchRate=\frac{MatchNum*2}{\left(TerNum1+TerNum2\right)}$$
    where MatchNum is the number of matching point pairs and *TerNum1* and *TerNum2* are the number of data points in the two point clouds. Regional maps with a matching rate below the threshold will be considered as unrelated.
  By selectively adding constraints based on the α and MatchRate thresholds, the graph structure remains sparse, ensuring optimization efficiency and good scalability as the map size increases.
(3) For each vertex already incorporated to the graph structure, examine its correlation with other regional maps. If the set conditions are satisfied, incorporate the regional map to the graph structure as a new vertex and establish an edge between the two vertices.
(4) Use the Gauss–Newton method to optimize and solve the graph structure.
(5) Adjust the regional maps based on the graph optimization results.
(6) Obtain the global map through the probabilistic fusion of regional maps.

## 4. Analysis of Regional Width

### 4.1. Effect of Area Width on System Performance

The proposed method partitions the global map into several relatively independent regions of size S*S, defined by the interval distance *S*. Within each region, the sensor data collected by the robot are fused to generate the corresponding regional map.

#### 4.1.1. Influence of Region Width on Regional Map Overlap Degree

**Figure 7:**
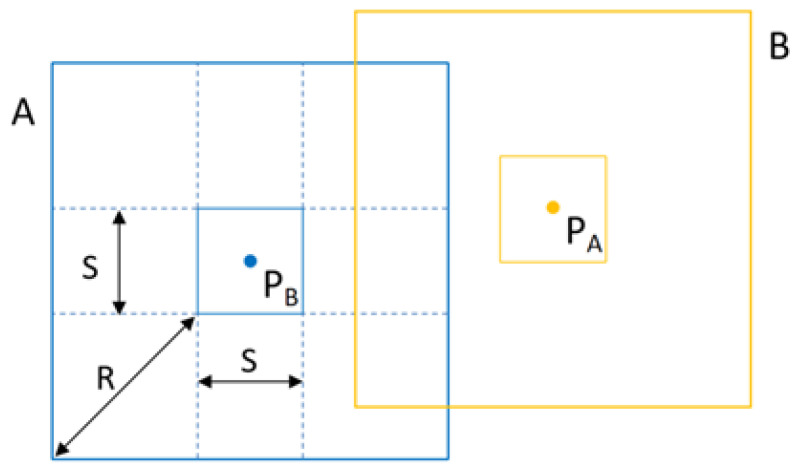
Overlapping range of regional maps.

The overlap range between regional maps is shown in Figure 7. The orange and blue thin solid line frames represent the ranges of Region A and B, respectively; $P_{A}\left(x_{A},y_{A}\right)$ and $P_{B}\left(x_{B},y_{B}\right)$ are the center coordinates of Region A and B, respectively; *S* is the region width; and *R* is the maximum measurement distance of the LiDAR sensor. The overlap degree of Regional Map A and Regional Map B is shown in Equation (12).

(12) $$Overlap\left(A,B\right)=\frac{\left(S+\frac{2R}{\sqrt{2}}-\left|x_{A}-x_{B}\right|\right)*\left(S+\frac{2R}{\sqrt{2}}-\left|y_{A}-y_{B}\right|\right)}{{\left(S+\frac{2R}{\sqrt{2}}\right)}^{2}}$$

where $S+\frac{2R}{\sqrt{2}}$ is the width of the regional map and $\left|x_{A}-x_{B}\right|$ and $\left|y_{A}-y_{B}\right|$ are the center distances between the two regions.

From Equation (12), it can be seen that for two adjacent regional maps with a known maximum measurement distance, the smaller the region width, the higher the overlap degree between the two regional maps. With a known region width, the farther the maximum measurement distance, the higher the overlap degree between the two regional maps.

#### 4.1.2. Influence of Region Width on Regional Map Storage Space


(13) $${RegNum}_{\max}=\left\lceil \frac{{MapWidth}_{x}}{S}\right\rceil *\left\lceil \frac{{MapWidth}_{y}}{S}\right\rceil$$

The maximum number of regional maps is shown in Equation (13), where S is the regional map width, ${MapWidth}_{x}$ and ${MapWidth}_{y}$ are the widths of the global map in the x and y directions, respectively, and ${RegNum}_{\max}\;$ is the maximum number of regional maps within the global map. From Equation (13), it can be seen that for a global map of the same size, the smaller the region width, the larger the maximum number of regional maps.

The maximum storage space required for regional maps is shown in Equation (14).

(14) $${StoreSize1}_{\max}={RegNum}_{\max}*{\left(S+\frac{2R}{\sqrt{2}}\right)}^{2}*\frac{unit}{w_{g}*w_{g}}$$

where ${RegNum}_{\max}$ is the maximum number of regional maps, ${\left(S+2R/\sqrt{2}\right)}^{2}$ is the area of the regional map, $w_{g}$ is the grid width, and unit is the storage space occupied by one grid cell. In this study, unit is taken as 2 bytes. From Equation (14), it can be seen that under the same conditions, the smaller the region width, the larger the maximum storage space required.

### 4.2. Region Width Test

As analyzed in Section 4.1, under the same conditions, a smaller region width results in a larger number and higher overlap degree between regional maps, which is beneficial for reducing system localization errors but also leads to an increase in storage and computational requirements. The number of regional maps within the robot’s working range can be obtained from Equation (15).

(15) $${Reg}_{num}=\left(\left\lfloor \frac{W_{x}}{S}\right\rfloor +1\right)*\left(\left\lfloor \frac{W_{y}}{S}\right\rfloor +1\right)$$

where $\left[W_{x},W_{y}\right]$ are the length and width of the test environment.

Substituting Equation (15) into Equation (14) gives the storage space required for the regional maps, as shown in Equation (16):

(16) $$\;Size=\left(\left\lfloor \frac{W_{x}}{S}\right\rfloor +1\right)*\left(\left\lfloor \frac{W_{y}}{S}\right\rfloor +1\right)*{\left(S+\frac{2R}{\sqrt{2}}\right)}^{2}*\frac{unit}{w_{g}*w_{g}}$$

**Figure 8:**
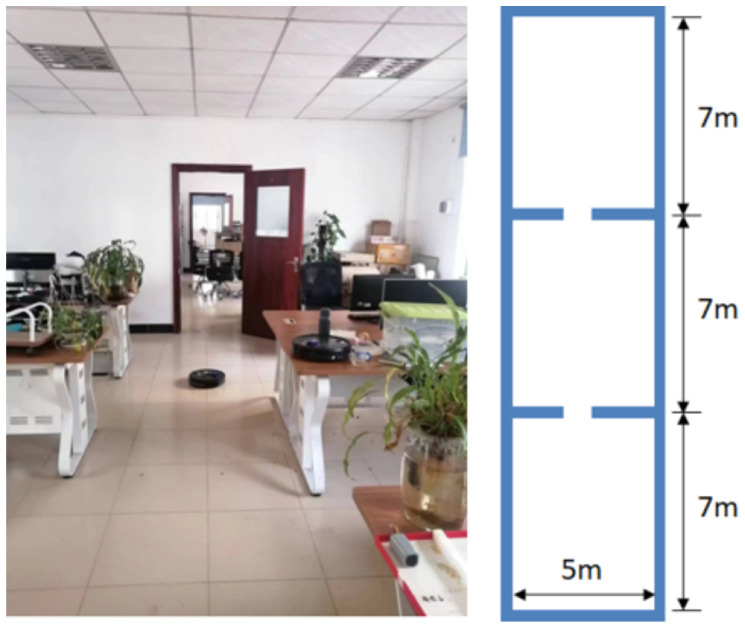
Test environment.

The test environment is shown in Figure 8; it can be seen that the test environment consists of three rooms, forming a rectangular space of 21 m × 5 m. The environment includes obstacles such as tables, chairs, and miscellaneous items.

**Figure 9:**
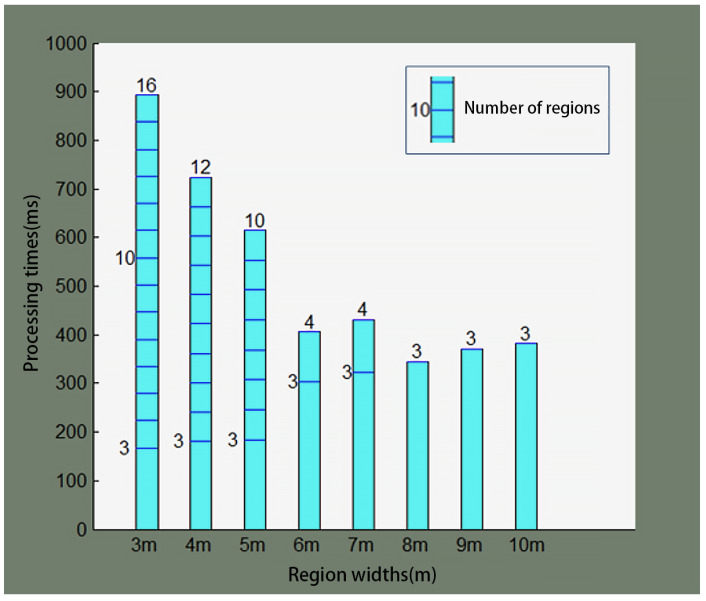
GO processing time at several region widths.

The graph optimization processing time under different region widths is shown in Figure 9. In the figure, each column corresponds to a different region width, the blue lines within the column represent the number of regional maps, and the number at the top of each column is the final number of regional maps.

Figure 9 shows that, in the same test environment, a larger region width produces fewer regional maps. As a result, the graph optimization processing time is shorter. As the robot continues to work, the number of regional maps increases, and so does the graph optimization processing time. In the initial phase of the robot’s operation, when the number of regional maps is less than 3, no graph structure can be established, and thus no graph optimization calculations are performed.

The system performance parameters under different region widths are statistically summarized in Table 1, where a smaller region width results in a larger number of generated regional maps, a smaller average localization error, but a longer graph optimization processing time and a larger storage space requirement for the regional maps. Based on the above tests, in general, the region width should be set to 4 m.

## 5. Experiment and Evaluation

### 5.1. Evaluation Standard

We designed a controlled validation environment (10 m × 10 m) containing characteristic structural features including 1.5 m wide corridors, modular workstation arrays, and orthogonal room partitions. This configuration emphasizes linear geometries and repetitive patterns that are characteristic of typical indoor environments targeted by our framework. To objectively evaluate the performance of the mapping method, the following metrics were designed: map storage space, point cloud data processing time, graph optimization processing time, and average localization error.

(1) Map Storage Space: The memory space required to store the global map, regional maps, and submaps.
(2) Point Cloud Processing Time ($T_{cloud}$): The time required for the robot to process the collected point cloud data, including data pre-processing, point cloud registration, map updates, and pose updates. A shorter point cloud processing time indicates higher algorithm efficiency and better real-time performance, and vice versa.
(3) Graph Optimization Processing Time ($T_{graph}$): The time required for the system to perform one graph optimization, including graph structure establishment, objective function solving, and node optimization. Graph optimization is performed when the robot leaves a region; this involves extensive computation and data processing. A shorter graph optimization processing time indicates higher algorithm efficiency, lower hardware load, and better real-time performance. An excessively long processing time may lead to system task congestion and high CPU utilization.
(4) Average Localization Error (${LocErr}_{ave}$): The distance error between the robot’s calculated position and the actual position in the environment. Pose samples are taken along the robot’s trajectory at regular intervals, and the localization error at these sample points is calculated. The average of the top 10% largest errors is taken as the average localization error, as shown in (17).
  (17) $${LocErr}_{ave}=\sum_{n=0}^{N*0.1-1}{err}_{n}/\left(N*0.1\right)$$
  where *N* is the number of pose samples and *err* is the array of localization errors sorted in descending order, with ${err}_{n}$ being the nth error. Smaller average localization errors indicate higher robot localization accuracy and better map-building performance; larger errors indicate lower accuracy and poorer performance.

### 5.2. Experiment and Results Analysis in Indoor Environment

**Figure 10:**
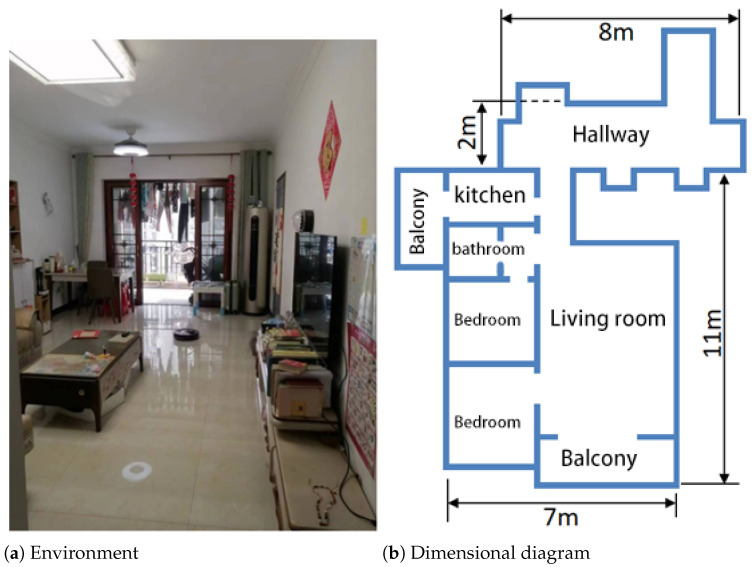
Indoor environment.

The evaluation focused on corridor navigation and workspace mapping, which are critical scenarios with dominant line features. The indoor environment is shown in Figure 10.

From Figure 10, it can be seen that the indoor environment is a residential setting, including a living room, bedrooms, balcony, kitchen, bathroom, and common hallways, with a total area of approximately 100 $m^{2}$. To enhance realism, the experiment was conducted in a real-world environment with normal human activities.

**Figure 11:**
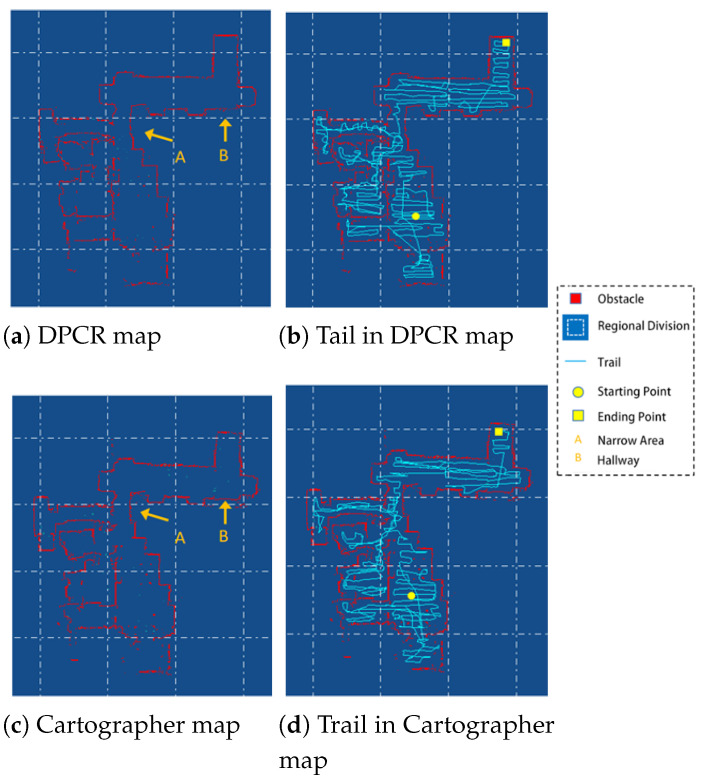
Experimental results in the indoor environment.

The experimental results of the two methods in the indoor environment are shown in Figure 11. Both methods used a grid width of $w_{g}=5$ cm and ran on the same robot platform.

From Figure 11a,c, it can be seen that the environment contains a relatively narrow area connecting two regions (indicated by arrow A), where the moving robot has difficulty simultaneously obtaining environmental information from both ends of the narrow area. Cartographer fuses point cloud information collected over a certain distance to generate submaps. Due to the limitations of the narrow area, the data correlation between submaps on either side of the narrow area is poor, leading to significant discrepancies in the global map in the regions on either side of the narrow area, as shown in region B in Figure 11c. In contrast, the proposed method employs a dual registration framework to fuse submaps into regional maps. This approach expands the detection range, enhances data correlation between maps, and avoids distortion in narrow areas, as shown in region B in Figure 11a.

From Figure 11b,d, it can be seen that to complete the floor cleaning task, the cleaning robot needs to traverse all accessible areas, resulting in dense trajectories. Cartographer store submaps for back-end graph optimization and global map generation. In practical robot applications, however, overlapping movement paths create redundancy between submaps, which wastes storage resources. In contrast, the proposed method stores regional maps for graph optimization, significantly reducing the number of stored maps and improving the issue of excessive information redundancy between different maps.

**Figure 12:**
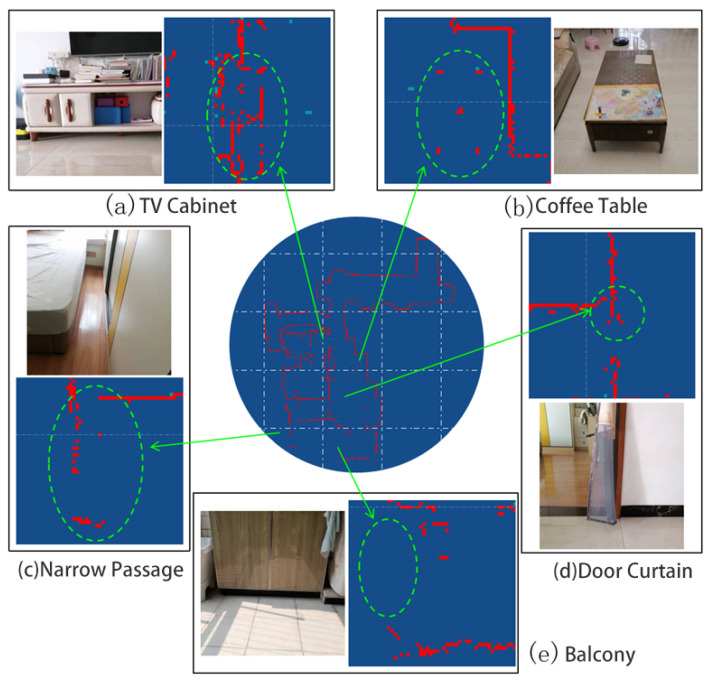
Details in the environment.

Several localized details in the map are shown in Figure 12.

Figure 12a shows a TV cabinet with a certain height between its bottom and the floor. This height is approximately the same as the robot’s LiDAR sensor. The sensor has a certain probability of detecting the wall behind the TV cabinet, and also a certain probability of detecting the cabinet itself. Therefore, this local environment is represented in the map as parallel, interlaced, and mutually complementary line segments.

Figure 12b shows a coffee table. There is a considerable distance between the bottom of the coffee table and the floor, allowing the robot to move through to the other side. This coffee table is represented in the map as five separate obstacles at certain distances from each other, corresponding to the five legs of the table.

Figure 12c shows a corner of a room, where a bed and a wardrobe form a narrow passage. The robot cannot enter this passage, nor can it detect the side of the passage near the bed. However, when the relative position between the sensor and the passage is appropriate, the sensor can detect one side deep in the passage and the side near the wardrobe.

Figure 12d shows a retracted door curtain. Although this curtain is narrow (about 20 cm) and semi-transparent, the robot can still accurately capture this obstacle, demonstrating the system’s ability to handle sensor noise.

Figure 12e shows a floor cabinet on the balcony. The part where the cabinet connects to the floor is a black, smooth-surfaced tile. Due to the surface characteristics of the object and the effect of sunlight, this section of the obstacle is missing from the map.

Table 2 shows the performance parameters of the two map-building methods in the indoor environment. As indicated in the table, under the same grid width and hardware platform conditions, DPCR-SLAM reduces the map storage space by 76.3%, the point cloud processing time by 55.8%, the graph optimization time by 77.7%, and the average localization error by 10.9% compared to Cartographer. The experimental results demonstrate the proposed method’s superior performance in structured environments, where the line-enhanced dual-registration mechanism effectively maintains geometric consistency across challenging features including narrow passages and repetitive architectural patterns. Moreover, although the 10.9% reduction in localization error may seem modest, it is meaningful because it achieves higher accuracy at a significantly lower system cost—a critical factor for commercial deployment.

## 6. Conclusions

This paper presents a mapping method for indoor robots based on line features and dual registration, addressing the issues of high computational complexity, high memory consumption, and suboptimal mapping in traditional methods. Our key innovation lies in an enhanced PLICP variant. This algorithm performs sequential point cloud alignment through hierarchical submap and regional map registration. It is further coupled with an efficient region-based graph optimization strategy. This architecture demonstrates remarkable efficiency gains. It achieves a 55.8% reduction in point cloud processing time and a 77.7% reduction in graph optimization time compared with existing methods. At the same time, it maintains higher mapping accuracy (10.9% lower localization error) while using 76.3% less memory, which means it can significantly reduce the production cost of indoor cleaning robots and is highly beneficial for industrial deployment.

However, with the advancement of sensor technology, recent works on 3D vision technologies for structural analysis [32] demonstrate the promising integration of multi-sensor fusion for precise environmental perception. Building on such advances while addressing challenges in sensor size, hardware resources, and power consumption, developing 3D LiDAR-based mapping methods for indoor cleaning robots will be the focus of our future work.

## Figures and Tables

**Table 1:** System performance under several region widths.

Region Width (m)	Number of Regions	Regional Map Storage (MB)	Processing Time (ms)	System Localization Errors (cm)	
				35 min	70 min
3	16	1.93	893	1.2	3.5
4	12	1.5	724	2	4.6
5	10	1	615	2.3	5.1
6	4	0.98	406	3.1	6.8
7	4	1.13	431	3.6	7.5
8	3	0.69	345	4.8	10.2
9	3	0.79	371	5.5	11.1
10	3	0.67	382	6.9	14.6

**Table 2:** Performance of the two mapping methods in the indoor environment.

	Storage (MB)	$T_{\mathrm{cloud}}$ (ms)	$T_{\mathrm{graph}}$ (ms)	${\mathrm{LocErr}}_{\mathrm{ave}}$ (cm)
DPCR-SLAM	2.2	115	870	4.1
Cartographer	9.3	260	3900	4.6
Improvement	76.3%	55.8%	77.7%	10.90%

## Data Availability

The authors declare that the data supporting the analysis are not available due to data privacy laws.
